# Low-molecular-weight-heparin versus a coumarin for the prevention of recurrent venous thromboembolism in high- and low-risk patients with active cancer: a post hoc analysis of the CLOT Study

**DOI:** 10.1007/s11239-019-01833-w

**Published:** 2019-03-11

**Authors:** Seth Woodruff, Agnes Y. Y. Lee, Marc Carrier, Guillaume Feugère, Paula Abreu, Joseph Heissler

**Affiliations:** 1 Pfizer Inc, New York, NY USA; 2 Vancouver Coastal Health and British Columbia Cancer Agency, University of British Columbia, Vancouver, BC Canada; 3 Department of Medicine, Ottawa Hospital Research Institute at the University of Ottawa, Ottawa, ON Canada; 4 Pfizer Canada Inc, Kirkland, QC Canada

**Keywords:** Active cancer, Risk factors, Anticoagulation, Recurrent thromboembolism, Bleeding

## Abstract

In patients with active cancer and acute venous thromboembolism (VTE), the low-molecular-weight-heparin (LMWH) dalteparin is more effective than vitamin K antagonist (VKA) in reducing the risk of recurrent venous thromboembolism (rVTE) without increasing the risk of bleeding. However, the relative benefit of LMWH versus VKA in patients with active cancer at high or low risk of rVTE and bleeding is unclear. This post hoc analysis used data from the CLOT study to explore the efficacy and safety of LMWH versus VKA in preventing recurrent thrombosis in high- and low-risk patients with active cancer. High-risk patients were defined by metastatic disease and/or antineoplastic treatment at baseline; low-risk patients presented with neither. Among high-risk patients, rVTE occurred in 25/318 (8%) (LMWH) versus 53/314 (17%) (VKA) (hazard ratio, 0.44; *p* = 0.001). No significant difference was detected in the rate of major or any bleeding. The 6-month mortality rate was 40% (LMWH) versus 41% (VKA). In low-risk patients, 2/20 (10%) (LMWH) had rVTE versus 0/24 (0%) (VKA) (hazard ratio, not estimable; *p* = 0.998). No significant difference was detected in the rate of major or any bleeding. The 6-month mortality rate was 20% (LMWH) versus 29% (VKA). In patients with cancer-associated thrombosis at high risk of rVTE and bleeding, the LMWH dalteparin was more effective than VKA in reducing the risk of rVTE without increasing the risk of bleeding. No difference in rate of rVTE or bleeding was observed between LMWH and VKA among low-risk patients.

## Highlights

- Patients with cancer-associated thrombosis are heterogeneous. Substantial differences in clinical risk factors between these patients may lead to different clinical outcomes despite therapeutic anticoagulation with the same agent.
- The relative benefit of low-molecular-weight-heparin (LMWH) in patients with cancer-associated thrombosis at different levels of risk for recurrent VTE (rVTE) and bleeding is unclear.
- This exploratory post hoc analysis of the pivotal CLOT trial compared the efficacy and safety of LMWH with that of vitamin K antagonist (VKA) in subgroups of patients with active cancer at high risk or low risk of recurrent thrombosis and bleeding.
- In patients with cancer-associated thrombosis at high risk of rVTE and bleeding, LMWH was more effective than VKA in reducing the risk of rVTE without increasing the risk of bleeding. However, no difference in efficacy or safety was observed between LMWH and VKA among low-risk patients.
- Future prospective studies are needed to further define the relative efficacy and safety of different anticoagulation treatments in patients with cancer-associated thrombosis and different levels of risk for rVTE and bleeding.

## Background

Current standard treatment for acute venous thromboembolism (VTE) in patients with active cancer is long-term low-molecular-weight-heparin (LMWH) [1, 2]. This recommendation is based primarily on the ‘Comparison of Low-Molecular-Weight-Heparin vs Oral Anticoagulant Therapy for the Prevention of Recurrent Venous Thromboembolism in Patients with Cancer (CLOT)’ study which showed an overall 52% relative risk reduction in the rate of symptomatic, recurrent venous thromboembolism (rVTE) over 6 months with the LMWH dalteparin versus oral vitamin K antagonist (VKA) therapy without an increased risk of bleeding [3]. This approach is effective in most patients, but patients with active cancer are heterogeneous. There are substantial differences in risk for rVTE and hemorrhagic complications because of differences in clinical risk factors [4]. Individual risk factors for rVTE in cancer cohorts include locally advanced or metastatic cancer, antineoplastic treatment, primary tumor site, and recent diagnosis of malignancy [2–6]. Key risk factors associated with an increased risk of major bleeding include metastatic disease, a recent bleeding event, and renal insufficiency [4]. Importantly, substantial differences in clinical risk factors between patients with cancer-associated thrombosis may lead to different clinical outcomes despite therapeutic anticoagulation with the same agent. As a result, the relative benefit of LMWH versus VKA in patients with cancer-associated thrombosis at high or low risk of rVTE and bleeding is unclear.

We performed an exploratory, post hoc analysis of the CLOT study to compare the efficacy and safety of the LMWH dalteparin versus VKA in subgroups of patients with active cancer at high risk or low risk of rVTE and bleeding.

## Methods

### Study design and population

A detailed description of the CLOT study design, population, treatment regimens, and outcome measures has been published previously [3]. The CLOT study was conducted in compliance with the ethical principles originating in, or derived from, the Declaration of Helsinki and in compliance with all International Conference on Harmonisation Good Clinical Practice Guidelines. All patients provided written, informed consent.

This exploratory post hoc analysis of data from the CLOT study compared the efficacy and safety of subcutaneous LMWH with that of oral VKA therapy in preventing rVTE in patients with active cancer at high or low risk of recurrent thrombosis and bleeding. High-risk patients were empirically defined as those with known metastatic disease and/or were receiving recent antineoplastic treatment (chemotherapy, radiation, and/or surgery 6 months prior to or at randomization) at baseline. These parameters, readily determined in clinical practice, are established independent risk factors for rVTE and bleeding during therapeutic anticoagulation [4]. Low-risk patients excluded high-risk patients as defined above, and therefore, included patients with neither metastatic disease nor antineoplastic treatment at baseline.

LMWH patients were administered 200 IU of dalteparin per kilogram once daily for 30 days, with a maximum daily dose of 18,000 IU. For the remaining 5 months, dalteparin was administered at a dose of approximately 150 IU per kilogram once daily. If the platelet count fell to 50,000–100,000 per microliter during treatment, the dalteparin dose was temporarily reduced.

Oral VKA patients initially received 200 IU of dalteparin per kilogram (maximal daily dose, 18,000 IU) once daily and, within 24 h, a vitamin K antagonist for 6 months. Dalteparin was discontinued after a minimum of 5 days and once the international normalized ratio (INR) remained between 2.0 and 3.0 for 2 consecutive days. All VKA doses were adjusted to reach a target INR of 2.5 (therapeutic range 2.0–3.0). Target INR was reduced to 2.0 (range 1.5–2.5) if the platelet count fell to 50,000–100,000 per microliter during treatment. In all patients, treatment was to continue for 6 months.

### Outcome measures

The primary efficacy outcome was the first episode of objectively diagnosed, symptomatic rVTE, including deep-vein thrombosis, pulmonary embolism, or both, during the 6-month study period in the intention-to-treat population (ITT; all randomized patients in the high- and low-risk patient subgroups).

Secondary outcomes included clinically overt bleeding during the 6-month study period (both any and major) and death. Major bleeding events were defined by an overt bleeding event associated with a fall in hemoglobin ≥ 2.0 g/dL, the need for a transfusion of ≥ 2 units of blood, involvement of a critical site (retroperitoneal, intracranial, intraspinal, intraocular, or pericardial areas), or an event leading to death. Any bleeding event also included all other overt hemorrhagic events that did not meet the criteria for classification as major bleeding. The safety analyses included patients who received at least one treatment dose.

All suspected rVTE and bleeding events were objectively reviewed and verified by an independent Central Adjudication Committee blinded to treatment allocation.

### Statistical analysis

Descriptive statistics were provided for baseline characteristics, tumor and VTE history, and risk factors. Time to first event analyses were conducted for the high-risk group using the Kaplan–Meier method and data were compared using the log-rank test with a 5% significance level. Cox proportional hazard regression models were employed to assess the effect of treatment (LMWH versus VKA) and other potential prognostic factors on VTE recurrence, bleeding, and death events in the high-risk group. Time to first event analyses and multivariate analyses were not conducted for the low-risk group due to the small sample size (*n* = 44). Competitive risk analysis for death was not performed in either group due to similar mortality rates between treatment arms [7]. Significance was set at the 5% level. No statistical adjustments for multiple testing were made.

## Results

### Study population

Baseline patient demographic and clinical characteristics stratified by risk (high/low) and treatment are shown in Table 1. Overall, 632/676 (93%) of patients in CLOT had metastatic disease and/or recent antineoplastic treatment at baseline (LMWH arm, 318; VKA arm, 314). Only 44/676 (7%) had neither metastatic disease nor recent antineoplastic treatment at baseline and were classified in the low-risk group (LMWH arm, 20; VKA arm, 24). In the high-risk subgroup, baseline demographics and clinical characteristics were similar between treatment arms. In the low-risk subgroup there was an imbalance in select baseline factors between treatment arms; however, no statistical comparisons were made because of the small number of patients.

**Table 1:** Baseline characteristics of high- and low-risk patient subgroups

	High-risk		Low-risk	
	Metastatic disease and/or antineoplastic treatment^a^		No metastatic disease, no antineoplastic treatment	
Parameter	Dalteparin, *n* = 318	VKA, *n* = 314	Dalteparin, *n* = 20	VKA, *n* = 24
Male, no. (%)	146 (45.9)	155 (49.4)	13 (65.0)	14 (58.3)
Age (years), mean (SD)	62.6 (11.33)	63.0 (12.65)	57.8 (15.71)	66.3 (11.64)
< 65 years, no. (%)	172 (54.1)	172 (54.8)	10 (50.0)	10 (41.7)
≥ 65 years, no. (%)	146 (45.9)	142 (45.2)	10 (50.0)	14 (58.3)
Weight (kg), mean (SD)	73.3 (15.31)	74.4 (16.50)	78.1 (17.55)	78.3 (20.45)
Creatinine clearance (mL/min), no. (%)				
Normal (CrCl ≥ 60)	228 (75.7)	207 (71.1)	17 (94.4)	18 (81.8)
Moderate RI (30 ≤ CrCl < 60)	64 (21.3)	78 (26.8)	1 (5.6)	4 (18.2)
Severe RI (CrCl < 30)	9 (3.0)	6 (2.1)	0 (0.0)	0 (0.0)
Tumor type, no. (%)				
Solid	283 (89.0)	294 (93.6)	15 (75.0)	14 (58.3)
Metastatic disease^b^	223 (78.8)	232 (78.9)	0 (0.0)	0 (0.0)
Hematological	35 (11.0)	20 (6.4)	5 (25.0)	10 (41.7)
Site of solid tumor cancer, no. (%)				
Bladder	10 (3.1)	18 (5.7)	0 (0.0)	1 (4.2)
Colorectal	51 (16.0)	50 (15.9)	1 (5.0)	1 (4.2)
Prostate	22 (6.9)	21 (6.7)	3 (15.0)	1 (4.2)
Brain	10 (3.1)	11 (3.5)	4 (20.0)	2 (8.3)
Lung	40 (12.6)	45 (14.3)	0 (0.0)	5 (20.8)
Testicle	1 (0.3)	2 (0.6)	0 (0.0)	0 (0.0)
Breast	58 (18.2)	49 (15.6)	1 (5.0)	0 (0.0)
Ovary	11 (3.5)	16 (5.1)	0 (0.0)	0 (0.0)
Uterus	12 (3.8)	2 (0.6)	1 (5.0)	0 (0.0)
Cervix	13 (4.1)	10 (3.2)	1 (5.0)	0 (0.0)
Pancreas	12 (3.8)	15 (4.8)	0 (0.0)	0 (0.0)
Other	43 (13.5)	55 (17.5)	4 (20.0)	4 (16.7)
Hematological tumor type, n (%)				
Non-Hodgkin’s lymphoma	19 (6.0)	10 (3.2)	3 (15.0)	5 (20.8)
Hodgkin’s lymphoma	5 (1.6)	2 (0.6)	0 (0.0)	0 (0.0)
Leukemia	7 (2.2)	1 (0.3)	1 (5.0)	3 (12.5)
Multiple myeloma	3 (0.9)	7 (2.2)	1 (5.0)	1 (4.2)
Performance status (ECOG)				
0	68 (21.4)	57 (18.2)	12 (60.0)	6 (25.0)
1	132 (41.5)	139 (44.3)	3 (15.0)	11 (45.8)
2	113 (35.5)	115 (36.6)	5 (25.0)	7 (29.2)
3	5 (1.6)	3 (1.0)	0 (0.0)	0 (0.0)
Recent antineoplastic treatment	266 (83.6)	259 (82.5)	0 (0.0)	0 (0.0)
Qualifying diagnosis				
DVT only	219 (68.9)	217 (69.1)	16 (80.0)	13 (54.2)
PE only	62 (19.5)	58 (18.5)	2 (10.0)	7 (29.2)
DVT and PE	37 (11.6)	39 (12.4)	2 (10.0)	4 (16.7)
Transient risk factors (last 12 weeks)^c^				
None	189 (59.4)	186 (59.2)	15 (75.0)	16 (66.7)
Hospitalization	78 (24.5)	85 (27.1)	3 (15.0)	7 (29.2)
Major surgery	57 (17.9)	64 (20.4)	5 (25.0)	3 (12.5)
Central venous catheter	46 (14.5)	40 (12.7)	0 (0.0)	0 (0.0)
Major trauma	3 (0.9)	4 (1.3)	0 (0.0)	0 (0.0)
Chronic risk factors^d^				
None	286 (89.9)	280 (89.2)	16 (80.0)	22 (91.7)
Chronic immobilization	26 (8.2)	26 (8.3)	2 (10.0)	2 (8.3)
Known thrombophilia	3 (0.9)	2 (0.6)	2 (10.0)	0 (0.0)
Strong family history of VTE	3 (0.9)	4 (1.3)	0 (0.0)	0 (0.0)
Paralysis or hemiparesis	3 (0.9)	3 (1.0)	0 (0.0)	0 (0.0)

*CrCl* creatinine clearance, *DVT* deep vein thrombosis, *ECOG* Eastern Cooperative Oncology Group, *PE* pulmonary embolism, *RI* renal impairment, *SD* standard deviation, *VKA* vitamin K antagonist, *VTE* venous thromboembolism

^a^Patients received antineoplastic treatment within 6 months prior to, or at, randomization

^b^Calculated as a percentage of solid tumors

^c^Patients may have > 1 transient risk factor

^d^Patients may have > 1 chronic risk factor

### VTE recurrence

**Fig. 1:**
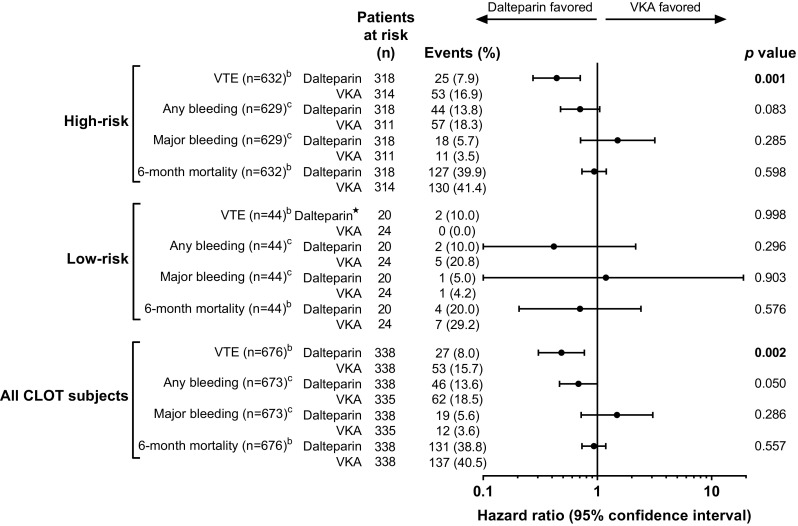
Comparison of treatment effects on the first VTE recurrence, first any bleeding, first major bleed, and death events in high- and low-risk patient subgroups. *Not estimable. ^a^Cox proportional model with treatment as covariate. ^b^Intention-to-treat patients. ^c^As-treated patients. Statistically significant *p* values (*p* < 0.05) are shown in bold text. *CI* confidence interval, *VKA* vitamin K antagonist, *VTE* venous thromboembolism, *High-risk* metastatic disease and/or antineoplastic treatment, *low-risk* no metastatic disease and no neoplastic treatment

First episodes of rVTE were determined in the ITT population according to treatment and risk subgroups (Fig. 1). Overall, 25/318 (8%) LMWH-treated patients and 53/314 (17%) VKA-treated patients in the high-risk group experienced ≥ 1 rVTE during the 6-month study period (hazard ratio [HR] 0.44; 95% confidence interval [CI] 0.273–0.708; *p* = 0.001). In contrast, 2/20 (10%) LMWH-treated patients and 0/24 (0%) VKA-treated patients in the low-risk group experienced ≥ 1 rVTE during the 6-month study period (HR; 95% CI, not estimable; *p* = 0.998).

**Fig. 2:**
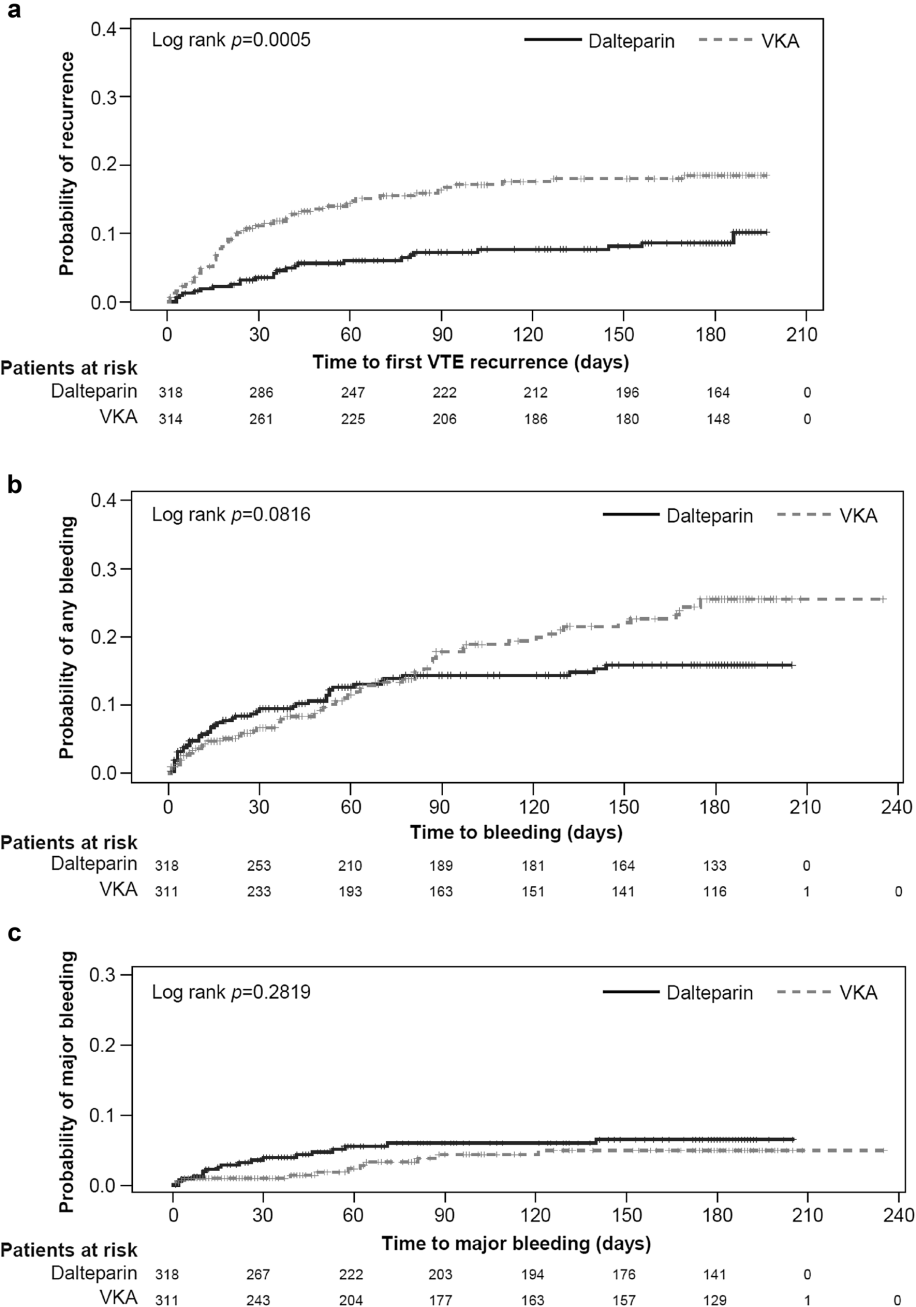
Kaplan–Meier estimates of the time to first VTE and bleeding events in high-risk patients. **a** Time to first VTE reccurence at 6 months^a^**b** Time to first any bleeding event at 6 months^b^**c** Time to first major bleeding event at 6 months^b^. Significance set at 5% and *p*-value calculated using log-rank test. ^a^ITT population. ^b^AST population. *AST* as-treated, *ITT* intention-to treat, *VKA* vitamin K antagonist, *VTE* venous thromboembolism

Kaplan–Meier curves showing time to first rVTE at 6 months for high-risk patients in the LMWH and VKA treatment groups are presented in Fig. 2.

**Fig. 3:**
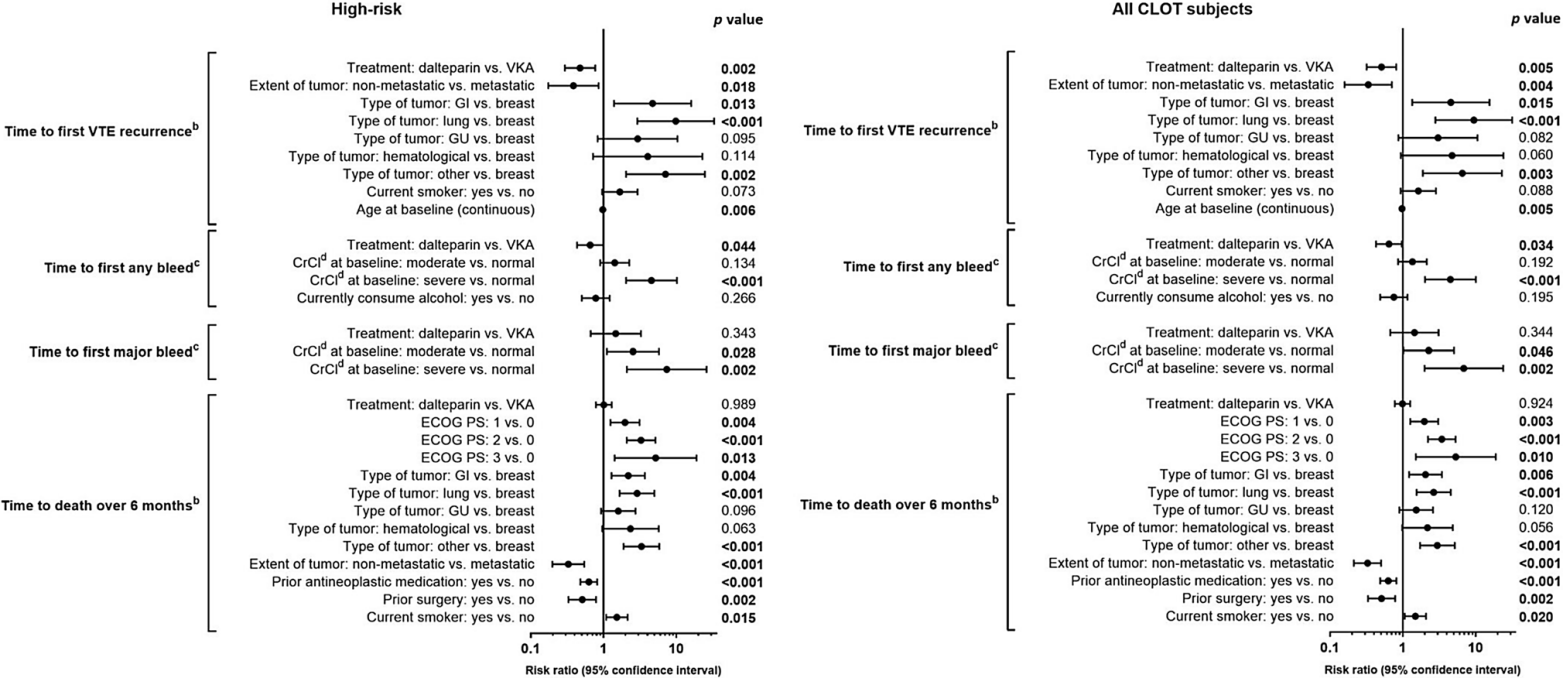
Time to first event data following adjustment for potential prognostic factors^a^ assessed at study entry. ^a^Prognostic variables applied to CLOT study. With the exception of treatment effect, only factors significant in a univariate analysis at the 0.10 significance level were considered in the process of building the final multivariate model. ^b^Intention-to-treat population. ^c^As-treated population. ^d^CrCl values were calculated using the Cockcroft-Gault formula. Normal: CrCl ≥ 60 mL/min; moderate impairment: 30 ≤ CrCl < 60 mL/min; severe impairment: CrCl < 30 mL/min. *High-risk* metastatic disease and/or antineoplastic treatment. Statistically significant *p* values (*p* < 0.05) are shown in bold text. *CI* confidence interval, *CrCl* creatinine clearance, *ECOG PS* Eastern Cooperative Oncology Group performance status, *GI* gastrointestinal, *GU* genitourinary, *VKA* vitamin K antagonist, *VTE* venous thromboembolism

In the high-risk group, Cox proportional hazards regression analyses demonstrated that treatment with LMWH lowered the risk of rVTE versus VKA. This reduction remained statistically significant after adjustment for other prognostic factors (risk ratio [RR] 0.47; 95% CI 0.293–0.764; *p* = 0.002; Fig. 3). Additionally, significant interactions between treatment group and risk factors were detected. The risk of rVTE was higher in patients with metastatic versus non-metastatic cancer (*p* = 0.018) and in younger patients versus older patients (*p* = 0.006). Notably, the risk of rVTE was not higher in patients with versus without recent antineoplastic treatment in the high-risk patient group.

### Bleeding events

First instances of any or major bleeding were determined in the as-treated population according to treatment and subpopulation (Fig. 1). The proportion of patients in the high-risk group experiencing ≥ 1 any bleeding episode was lower in the LMWH arm than in the VKA arm: 44/318 (14%) versus 57/311 (18%), respectively, but this difference was not statistically significant (HR 0.71; 95% CI 0.476–1.047; *p* = 0.083). The proportion of patients in the low-risk group experiencing ≥ 1 any bleeding event was also lower in LMWH-treated patients (2/20 [10%]) versus VKA-treated patients (5/24 [21%]). This difference was not statistically significant (HR 0.42; 95% CI 0.080–2.160; *p* = 0.296).

A greater proportion of high-risk patients experienced ≥ 1 major bleeding episode while receiving LMWH (18/318 [6%]) than VKA (11/311 [4%]). However, between-treatment differences were not statistically significant (HR 1.51; 95% CI 0.711–3.188; *p* = 0.285). The rate of major bleeding episodes in the low-risk group was similar between LMWH- and VKA-treated patients (1/20 [5%] vs. 1/24 [4%], respectively).

Kaplan–Meier curves estimating time to first any or major bleeding event over 6 months for high-risk patients are presented in Fig. 1. The cumulative probability of any or major bleeding event over 6 months for LMWH versus VKA was not statistically significant (2-sided log-rank test: any bleeding event, *p* = 0.082; major bleeding event, *p* = 0.282).

Cox proportional hazards models for the high-risk group demonstrated a statistically significant lowered risk of any bleeding event for LMWH versus VKA after adjustment for other prognostic factors for bleeding (RR 0.65; 95% CI 0.431–0.989; *p* = 0.044; Fig. 3). Additionally, the risk of any bleeding was significantly lower in patients with normal renal function versus severe renal impairment (creatinine clearance [CrCl] < 30 mL/min) (*p* < 0.001), a finding comparable with the full CLOT study [8]. The larger proportion of high-risk patients experiencing ≥ 1 major bleeding event with LMWH versus VKA remained non-significant when the model was adjusted for other prognostic factors for bleeding (RR 1.47; 95% CI 0.664–3.243; *p* = 0.343). The risk of major bleeding events was higher in patients with moderate (30 < CrCl < 60 mL/min) (*p* = 0.028) or severe renal impairment (*p* = 0.002) versus normal renal function. Neither metastatic disease alone nor recent antineoplastic treatment alone increased the rate of any or major bleeding events in this high-risk patient group.

### Death events

The death rate among high-risk patients during the 6-month study period was 127/318 (40%) in LMWH-treated patients and 130/314 (41%) in VKA-treated patients. Among low-risk patients, 6-month mortality was lower: 4/20 (20%) in the LMWH group and 7/24 (29%) in the VKA group.

In the high-risk group, Cox proportional hazards models, adjusted for other factors prognostic for death, demonstrated similar 6-month mortality rates between LMWH and VKA groups (RR 1.00; 95% CI 0.781–1.285; *p* = 0.989; Fig. 3). Metastatic disease was found to increase 6-month mortality rates in this high-risk patient group.

## Discussion

To our knowledge, this exploratory, post hoc analysis is the first to study the efficacy and safety of LMWH versus VKA for the treatment of acute VTE in patients with active cancer at high or low risk of rVTE and bleeding complications. High-risk patients, 93% of the total CLOT population, were defined as having metastatic disease and/or receiving recent antineoplastic treatment at baseline, while low-risk patients had neither. These definitions were empirically derived, based on prior reports and clinical experience that metastatic disease and active cancer therapy are risk factors for recurrent thrombosis and bleeding. Our analysis showed that LMWH treatment for up to 6 months in high risk patients was more effective than VKA in reducing the risk of recurrent thromboembolism without increasing the risk of bleeding. At 6 months, the overall risk of recurrent thrombosis and major bleeding was 12% and 5%, respectively. Importantly, the 8% rate of rVTE at 6 months in the high-risk LMWH group of the CLOT study is consistent with the rate reported recently for LMWH in the DALTECAN Study [9].

Among high-risk patients, there was a higher risk of rVTE in patients with metastatic versus non-metastatic cancer, which supports the empirical use of baseline metastatic cancer to define high-risk patients. Interestingly, the risk of rVTE was not higher in patients who received recent antineoplastic treatment versus those without such treatment. Together, these findings suggest that baseline metastatic disease may contribute more to increased risk of recurrent thrombosis than recent antineoplastic treatment in high-risk patients. Neither metastatic disease nor recent antineoplastic treatment was found to increase the rate of any or major bleeding events in the high-risk patient group. As a result, the risk of hemorrhagic complications under therapeutic anticoagulation may be more dependent on other risk factors than baseline metastatic disease or antineoplastic treatment. Recent findings from randomized trials comparing dalteparin with a direct oral anticoagulant (DOAC) [10, 11], indicate that choice of anticoagulant is an important predictor of bleeding, and that DOAC therapy may not be the optimal treatment for cancer-associated thrombosis among those patients with risk factors for bleeding.

Treatment with LMWH in patients with active cancer at low risk of rVTE and bleeding complications showed similar rates of rVTE and bleeding with VKA. These results should be interpreted with caution due to the small number of subjects and imbalance of prognostic factors (eg, age, tumor type and site, hospitalization) in the low-risk treatment group. However, our results are consistent with the CATCH trial, which showed that the LMWH tinzaparin was not more effective than VKA in preventing rVTE [12]. Although the CATCH trial used eligibility criteria similar to CLOT, it had fewer patients with metastatic disease and/or receiving anticancer therapy and better overall survival. Similar findings have also been reported in previous trials comparing LMWH and VKA for secondary prophylaxis of VTE in patients without cancer who are at low risk of recurrent events and hemorrhagic complications [13, 14]. Consequently, patients with less advanced cancer or better prognosis who are at lower risk of rVTE may not experience the same benefit with LMWH as those with metastatic malignancies.

Data to date show that there are substantial and clinically relevant differences in baseline patient characteristics and prognosis among various clinical trials (Table 2). This heterogeneity is an important consideration for cross-study comparisons and network meta-analyses in determining optimal anticoagulant therapy. For example, compared with high-risk CLOT patients, fewer patients with cancer in the DOAC-VKA [15–17], CATCH [12], and Hokusai VTE Cancer (edoxaban versus dalteparin) [10] trials had metastatic disease at baseline. Fewer patients in both the CATCH and Hokusai VTE Cancer trials had recent antineoplastic therapy at baseline or an Eastern Cooperative Oncology Group performance status of 2. Most importantly, all-cause mortality was lower in patients with cancer in DOAC-VKA trials, CATCH, and Hokusai VTE Cancer trials than in high-risk patients enrolled in CLOT. Notably, 6-month overall mortality was 26% in Hokusai VTE Cancer but 41% in high-risk CLOT. Despite a decade gap between these two trials, it is unlikely that differences in cancer therapeutics alone are responsible for the observed difference in mortality. As a result, patients with cancer in the DOAC-VKA, CATCH, and Hokusai VTE Cancer trials had better prognosis, with lower thrombotic and hemorrhagic risks, than overall and high-risk patients in CLOT. These findings are a strong reminder that extrapolation of clinical outcomes from one patient cohort to another may not be valid and should be avoided.

**Table 2:** Comparison of baseline characteristics and mortality rates in trials/subgroup analyses investigating DOAC or LMWH for the treatment of VTE in patients with cancer

Parameter	Rivaroxaban versus VKA [15]	Dabigatran versus VKA [16]	Edoxaban versus VKA [17]	Tinzaparin versus VKA (CATCH) [12]	Edoxaban versus dalteparin (Hokusai VTE Cancer) [10]	Dalteparin versus VKA (CLOT) [3]	Rivaroxaban versus dalteparin (SELECT-D) [11]
Trial/subgroup analysis							
Patients with cancer at baseline, *n*^a^	462	221	771	900	1046	676	406
Patients with metastatic disease at baseline, *n* (%)	101 (22)	28 (13)	46 (6)	492 (55)	554 (53)	455 (67)	236 (58)
Patients receiving antineoplastic therapy at baseline, *n* (%)	NR	NR	NR	476 (53)	757 (72)	525 (78)	282 (69)
ECOG PS = 2	NR	NR	NR	209 (23)	247 (24)	240 (36)	95 (23)
Mortality rate^b^, *n* (%)	74 (16)^c^	32 (14)^d^	80 (10)^c^	288 (32)^d^	267 (26)^d^	266 (40)^d^	104 (26)^d^

*DOAC* direct oral anticoagulant, *ECOG PS* Eastern Cooperative Oncology Group performance status, *LMWH* low-molecular-weight-heparin, *NR* not reported, *VKA* vitamin K antagonist, *VTE* venous thromboembolism

^a^The sum of solid and hematological malignancies

^b^Calculated as a percentage of the intention-to-treat population

^c^12-month mortality rate

^d^6-month mortality rate

Overall, our results suggest that patients with cancer-associated thrombosis have different levels of risk for rVTE and bleeding which can lead to different clinical outcomes despite therapeutic anticoagulation with the same agent. As a result, clinicians must be mindful of patient and cancer characteristics when considering the choice of anticoagulant therapy. Our results, combined with recent clinical trials, suggest that patients with active cancer at high risk of rVTE and, particularly, bleeding are likely to benefit more from therapeutic anticoagulation with LMWH, whereas lower-risk patients can choose among LMWH, DOAC, and warfarin. Individual patient benefit-risk assessment and preference should be essential components of the therapeutic decision.

Interpretation of this post hoc analysis has limitations. First, CLOT did not stratify patients by specific risk factors, and was not powered to detect between-treatment differences in subgroup analyses [3]. However, we used a priori documented baseline characteristics that have been established as important risk factors for thrombosis and bleeding. Second, we did not examine other risk factors such as biomarkers. Our approach was to use easily identifiable features for simple risk stratification. Finally, patient numbers in the low-risk non-metastatic disease/non-antineoplastic treatment subgroup were small, thus limiting the validity and value of our findings for this subgroup.

## Conclusions

Among patients with active cancer and acute VTE at high risk of recurrent thrombosis and bleeding, long-term self-injection of LMWH was more effective than oral VKA in reducing the risk of rVTE without increasing the risk of bleeding. In contrast, no difference in rate of rVTE or bleeding was observed between LMWH and VKA in low-risk cancer patients.
